# Long-term warming and human-induced plankton shifts at a coastal Eastern Mediterranean site

**DOI:** 10.1038/s41598-023-48254-7

**Published:** 2023-11-29

**Authors:** K. Kalloniati, E. D. Christou, A. Kournopoulou, J. A. Gittings, I. Theodorou, S. Zervoudaki, D. E. Raitsos

**Affiliations:** 1 Department of Biology, National and Kapodistrian University of Athens, 15772 Athens, Greece; 2 Institute of Oceanography, Hellenic Centre for Marine Research (HCMR), 19013 Anavyssos, Attica, Greece

**Keywords:** Phenology, Macroecology

## Abstract

Plankton are key ecological indicators for assessing the impacts of human-induced pressures like climate change and waste-water discharge. Here, 26 years (1988–2015) of biweekly in-situ chlorophyll-a concentration, mesozooplankton biomass and remotely-sensed sea surface temperature (SST) data are utilized to investigate long-term changes of plankton biomass and timing of growth (phenology) in relation to warming, in a coastal region of the Saronikos Gulf (Aegean Sea). A Waste-Water Treatment Plant (WWTP) was established in 1995, leading to decreased nutrient concentrations circa 2004. Overall, the results indicate an interplay between warming and changes in ecological status. During higher nutrient input (1989–2004), a temporal mismatch between zooplankton and phytoplankton, and a positive zooplankton growth—SST association, are evident. Conversely, in the warmer, less mesotrophic period 2005–2015, an earlier timing of zooplankton growth (related to copepod abundance) synchronizes with phytoplankton growth, including a secondary autumn growth period. Concurrently, an abrupt negative interannual relationship between SST and mesozooplankton, and a summer biomass decrease (linked with cladoceran abundance) are observed. This work provides evidence that current warming could alter plankton abundance and phenology in nearshore Eastern Mediterranean ecosystems, suggesting shifts in plankton community composition that could trigger potential cascading effects on higher trophic levels.

## Introduction

Phytoplankton are responsible for nearly half of global net primary production, constituting the principal energy source for marine ecosystems, through carbon fixation^1^. Their abundance and composition all play a significant role in global biogeochemical and energy cycles, climate regulation, and the biodiversity of higher trophic levels^2–5^. Similarly, zooplankton are an essential component of marine ecosystems through their control on phytoplankton biomass (via grazing) and the direct transfer of energy through the marine food web^6–9^. Zooplankton also benefit both the microbial community and phytoplankton by contributing to the regeneration of nutrients through excretion^10–12^.

Similarly to plankton abundance, phenology (timing of growth) plays an important role in shaping ecological interactions, food-web structures and ecosystem functioning^13–15^. Investigating the phenological coupling or decoupling between phytoplankton, zooplankton, and higher trophic levels can provide further knowledge on the ecosystem repercussions of these changes^13,15,16^. For instance, according to the match-mismatch hypothesis^13^, climate-driven shifts in plankton phenology can negatively impact consumers’ survival, due to the temporal asynchrony between consumer demand (i.e., larvae) and food availability^17–19^. Such consequences can be detrimental to both ecosystems and ecosystem services, by setting off a chain reaction of trophic mismatch in the food-web and by jeopardizing the recruitment success of commercially important species. Thus, metrics characterizing plankton phenology (including the timings of growth initiation, maximum amplitude, termination and duration) have been proven valuable for understanding the response of marine ecosystems to climate-driven changes^20–26^.

Increasing trends in anthropogenic greenhouse gas emissions over the past 50 years have contributed to the rapid warming of the atmosphere and oceans^27,28^. Temperature is considered one of the most important factors that modulates both directly (i.e., metabolic rates, reproduction, respiration, etc.) and indirectly (through physical mechanisms, such as vertical stratification) plankton phenology, abundance, competition, prey-predator interactions and geographical distribution^29^. Sea surface temperature (SST) is an important environmental proxy for the manifestation of climate change and its impact on the state of the oceans^30,31^. In regions that have been characterized as hot-spots for climate change, such as the Mediterranean Sea, the aforementioned impacts on marine ecosystems are expected to be more pronounced^32–35^.

In addition to rapid oceanic warming, other environmental pressures may alter the trophic interactions of planktonic species. Anthropogenic pollution can significantly affect plankton productivity by increasing the concentrations of dissolved nutrients in aquatic environments. Different sources of human-induced marine pollution can lead to deviations of the stoichiometric ratios of nutrients (N, P, Si), thus, disturbing optimal plankton growth and transforming the trophic status and water quality of a region^36,37^. Excessive nutrient loading is often associated with high levels of phytoplankton biomass and shifts in the community structure of planktonic primary producers^38,39^. The deterioration of ecological conditions, in these cases, could be evident through the occurrence of harmful algal blooms, the reduced ability of grazing control by zooplankton, and/or the decrease in water column transparency and the depletion of deep-water oxygen^40^.

The Eastern Mediterranean Sea has been described as one of the most oligotrophic areas in the world^41–46^. Although oligotrophic conditions dominate the open waters of the Mediterranean Sea, several overpopulated coastal zones lead to elevated nutrient concentrations in adjacent coastal marine ecosystems^32,36,47–49^. Excess nutrient loading, either from domestic and industrial effluents, or from fertilizers, farming and river fluxes, has transformed numerous oligotrophic near-shore waters into more mesotrophic or even eutrophic ecosystems^50–56^.

The exposure of coastal waters to human pressures (i.e., sea surface warming, excess nutrient input) and a complexity of physical and biological processes can lead to a high level of spatial and temporal variability in plankton abundance and phenology^57^. The interannual variability of these shaping factors makes it difficult to unravel the underlying mechanisms that control the regularity of observed plankton signals^57^. In addition, short-term studies or studies based on low sampling frequency are usually proven inadequate for elucidating plankton seasonal patterns and interannual fluctuations. A reliable tool to tackle the above issues is the construction of long-term data series, as they can provide further insights on plankton seasonal cycles and multi-annual trends^17,57–64^. Moreover, the use of long-term in-situ measurements of various physicochemical, biological or hydro-climatic indicators—observations that are available in the Eastern Mediterranean Sea—enables the investigation of ways in which changes in environmental or anthropogenic factors drive potential shifts in plankton phenology and community composition^65–68^.

With the synergistic use of a unique long-term (1988–2015) biweekly in-situ dataset of mesozooplankton biomass and chlorophyll-a concentration, and concurrent remotely-sensed SST observations, this study aims to investigate the interannual variability and phenological shifts in phyto- and zooplankton biomass within a warmer, anthropogenically-impacted coastal area of the Aegean Sea (Saronikos Gulf). In addition, zooplankton abundance data from a nearby offshore station are utilized to further examine variations in biomass in relation to the two prominent zooplankton groups, copepods and cladocerans.

## Methods

### Study area

**Figure 1:**
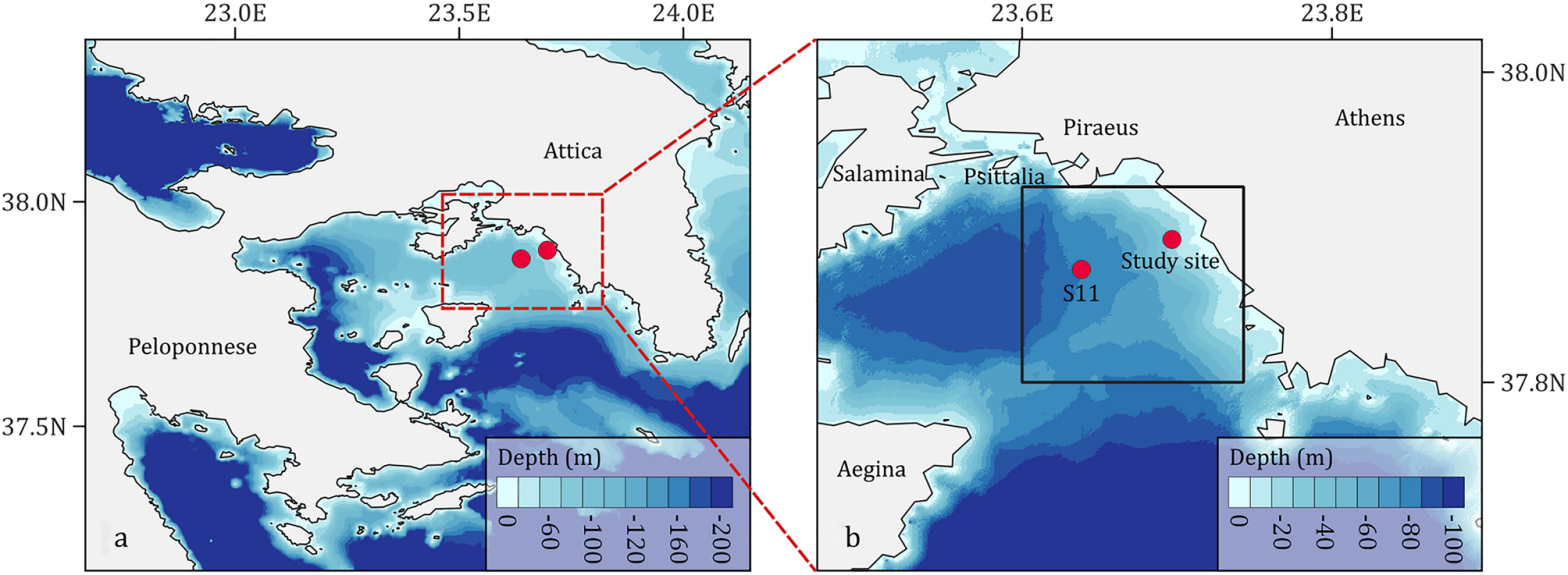
Map of the Saronikos Gulf (**a**) and of the Eastern Inner part of Gulf (**b**). The red circles mark the nearshore site and the S11 monitoring station, from which the in-situ samples were taken. The black rectangle corresponds to the area that was selected for the analysis of the remotely-sensed SST dataset.

The study area comprises a nearshore region of the Saronikos Gulf in the Aegean Sea (Fig. 1). The Inner Saronikos Gulf is a shallow area where depths do not exceed 100 m. It is enclosed by the Salamina and Aegina islands and connects with the coast of Attica and the open sea (Aegean Sea) at its northeast and southeast boundaries respectively^69^. The Saronikos Gulf is considered to be one of the most impacted gulfs by human activity, in Greece, as it receives the domestic and industrial wastes of approximately 4 million inhabitants of Athens and its outskirts^70^. Moreover, Piraeus harbor, located in the northwestern limit of the Inner Gulf, constitutes one of the busiest shipping areas in the Eastern Mediterranean Sea^71^.

Sewage discharge is considered to be the main source of pollution in the Saronikos Gulf, causing eutrophication in coastal regions close to the sewage outfall^50,72^. Before 1995, the untreated effluents of Athens were discharged into the surface waters of Keratsini Bay^70^. Between 1995–2004, sewage was transferred to the Psittalia Waste Water Treatment Plant (WWTP), where it underwent primary treatment before being discharged through multiport diffusers at a depth of 63 m and at a distance of ~ 2 km from the coast^70^. The operation of the secondary stage of treatment in WWTP started at the end of 2004, leading to a significant decrease in nutrient concentrations and organic load^73–75^. As a result, the ecological status of the Saronikos Gulf showed strong indications of improvement^70,76,77^. Thus, our analysis is henceforth divided into two study periods, P1 (1988–2004) and P2 (2005–2015).

### Study site

#### In-situ data

A total of 631 field samples were collected biweekly from a coastal site (12 m depth) in the Inner Saronikos Gulf (37.89N, 23.69E), over a 26-year period between November 1988 and April 2015 (Fig. 1b). The intervals between successive samplings ranged from 12 to 17 days but were treated as constant (14 days) for the purpose of the study. Mesozooplankton samples were collected, during daytime, by oblique hauls from the bottom to the surface, using a 200 µm (WP2) net equipped with a Hydrobios flowmeter^59^. After each haul and careful net rinsing, the contents of the cod end were immediately preserved in 4% buffered formalin. In the laboratory, zooplankton samples were obtained, after being divided with a Folsom plankton sample splitter. Within one week from the initial collection, the total zooplankton biomass was measured using the dry-weight method^78^.

Temperature, chlorophyll-a (Chl-a) and salinity were measured from samples taken at 1, 5 and 10 m, using a 2L Hydrobios water sampler equipped with a Hydrobios thermometer, and the mean values for the water column were estimated. Salinity measurements were conducted with the use of a Guildline 8400B Autosal salinometer. Chl-a was determined by fluorometric measurements of acetone extracts^79^. Approximately 1L of seawater was filtered through Whatman GF/F filters (0.7 μm pore size) and frozen at − 20 °C until analysis. The fluorescence of the extracted Chl-a was measured using a Turner AU-10 fluorometer, and Chl-a data were used as a proxy for phytoplankton biomass.

Biweekly values of Chl-a and zooplankton biomass were used for the computation of the annual averages and the biweekly climatologies over each study period (P1 & P2). To calculate the difference between the two periods, the biweekly climatology values of P1 were subtracted from those of P2, for each dataset.

#### Sea surface temperature remote sensing data

Daily (nighttime), optimally interpolated (L4), satellite-based estimates of the foundation SST were obtained from the CMEMS reprocessed Mediterranean SST dataset (https://doi.org/10.48670/moi-00173), which has a 0.05° × 0.05° (~ 5.5 km^2^) spatial resolution, available from 1982—present. The level 4 SST data were averaged over: a pixel nearest to the study site (37.89N, 23.71E) and a region of the inner Gulf (Fig. 1b, black rectangle), surrounding the study site and nearby station S11 (37.8, 37.92N, 23.60, 23.74E). The satellite-derived SST data sets were compared with the in-situ SST time series quantitatively, using Pearson’s correlation coefficient (r)^80,81^. Both daily match-ups and monthly mean values were used for the correlation analysis (Supplementary Fig. S1). A very high, significant correlation coefficient (r > 0.99, *p* < 0.00001—Supplementary Fig. S1) was found for every comparison between in-situ data and the satellite-derived SST product. In parallel, the daily match-up between the 1 pixel above the study site and the inner Gulf region resulted in an exceptionally high Pearson’s correlation coefficient (r = 0.9999, *p* < 0.00001—data not shown), indicating homogeneity in sea surface temperature at the Inner Gulf region. The higher-resolution (daily values) and gap-free remotely-sensed SST data set averaged over the inner Gulf region (Fig. 1b, black rectangle) was considered to be the most representative of the inner Saronikos Gulf, both temporally and spatially, and was finally selected for the analysis of SST data, following the finding of a remarkable agreement with the in-situ time series (Supplementary Fig. S1).

#### Phenology metrics

Plankton growth periods (phenological metrics of timings of initiation, peak and termination, and the duration) were calculated using a threshold-based phenology approach^82–85^. First, gap-free time series were calculated by applying a linear interpolation method and biweekly climatologies were calculated for both study periods (i.e., P1: 1988–2004 and P2: 2005–2015). A threshold criterion was defined as the long-term median, plus 5%^24,83^, allowing us to detect two phytoplankton growth periods. The choice of threshold depends on the magnitude of the seasonal peak and the type of analysis. In our case, selecting a higher threshold would result in the identification of just one growing period, since it would exceed the maximum value of one of the growth periods. Next, anomalies were computed by subtracting the threshold criterion from the climatologies and the cumulative sum of anomalies was calculated. The phenology metrics were then determined using the gradient of the cumulative sum, smoothed with a Gaussian filter. The gradient's rise above or fall below zero determines the timings of initiation and termination, respectively, while the number of two-week composites between the initiation and termination determines the total duration. Note that, in a given period, shorter fluctuations (< = 4 weeks) of plankton biomass that exceeded the threshold, although detected by the phenology algorithm, were not considered as plankton growth periods. Finally, the growth peak was determined, in accordance with the climatologies, as the time at which the maximum signal (Chl-a or biomass) was reached.

### Statistical analyses

Pearson’s correlation coefficient was calculated for the determination of the long-term trends and interannual relationships between zooplankton biomass, Chl-a, SST, and salinity, based on annual mean values or seasonal averages, for each of the study periods (P1: N = 16, P2: N = 10) and for the total time series (N = 26). All data analyses were executed in Python 3.9.

### Station S11

#### In-situ zooplankton abundance data

Zooplankton abundance data for the two dominant mesozooplankton groups, copepods and cladocerans^86–88^, were acquired from a permanent sampling station (S11, 37.876667N, 23.641667E) located in the inner Saronikos Gulf. At a distance of ~ 5 km offshore, station S11 is the nearest monitoring station to the nearshore sampling site of this study and has a total depth of 78 m. Sampling was carried out through vertical hauls with a 200 μm WP2 net, from the near-bottom to the surface. Overall, 85 mesozooplankton samples were used for the calculation of total copepod and cladoceran abundance between February—1987 and August—2009. Data were processed on an annual basis and a separation was made between 1988 and 2004 (P1) and 2005–2009 (P2), and between the detected two main growth periods of zooplankton in winter (February and March) and summer (June and July).

## Results

### Long-term interannual variability

**Figure 2:**
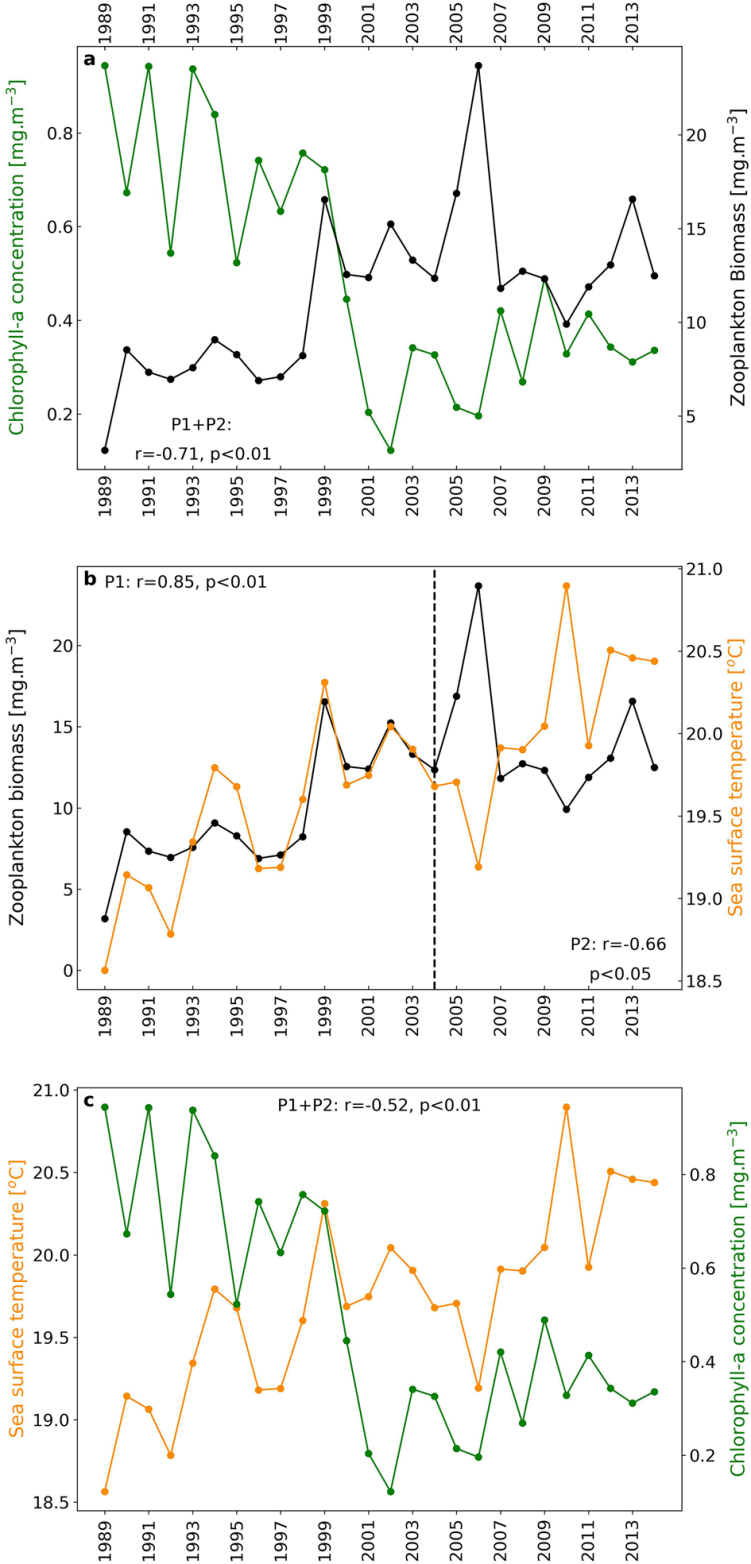
Interannual variability of zooplankton biomass versus Chl-a (**a**) and SST (**b**), and of Chl-a in relation to SST (**c**) for the period 1989–2014, based on annual mean values. The correlation between zooplankton biomass and Chl-a was significantly negative for the whole period (1989–2014), and the correlation between zooplankton biomass and SST was significantly positive for P1 and significantly negative for P2. Chl-a and SST were negatively correlated throughout the time series.

To examine the interannual variability of Chl-a, zooplankton biomass and SST, as well as their relationships, annual time series of each dataset were produced (Fig. 2), and the corresponding correlation coefficients were calculated for the entire time series, as well as for the periods P1 (1989–2004) and P2 (2005–2014). The results of all the statistical relationships are summarized in Supplementary Table S1. A significant, negative correlation between Chl-a and zooplankton biomass was evident throughout the whole time series (r = − 0.71, N = 26, *p* < 0.01, Fig. 2a), as well as during each separate study period (Supplementary Table S1). During P1, both SST and zooplankton biomass exhibited an increasing trend and a clear significantly positive correlation (r = 0.85, N = 16, *p* < 0.01, Fig. 2b). In contrast, during P2, this relationship became significantly negative (r =  −0.66, *p* < 0.05, Fig. 2b). Finally, SST and Chl-a concentration were negatively correlated throughout the whole time series, (r = − 0.52, *p* < 0.01, Fig. 2c), though this relationship was statistically significant only during P1 (r = − 0.51, *p* < 0.05, Supplementary Table S1).

Overall, zooplankton biomass interannual variability exhibited an increasing trend (r = 0.62, *p* < 0.01, Supplementary Fig. S2), which was significant during P1 (r = 0.83, *p* < 0.01, Supplementary Table S1) as opposed to P2 (r = − 0.33, *p* > 0.05, Supplementary Table S1). Conversely, Chl-a showed a strong, statistically significant decreasing trend (r = − 0.74, *p* < 0.01, Supplementary Fig. S2), during P1 (r = − 0.80, *p* < 0.01, Supplementary Table S1), whilst P2 was characterized by a slight, increasing Chl-a trend. Finally, SST was characterized by an increasing trend throughout the time series (r = 0.79, *p* < 0.01, Supplementary Table S1).

To further examine the reversal in the interannual relationship between zooplankton biomass and SST (Fig. 2b), seasonal averages (winter, spring, summer, autumn) were computed and the correlation coefficient (r) was calculated for P1 and P2, separately (Supplementary Fig. S3). During P1, the correlation between zooplankton and SST was positive for every season, but only statistically significant for winter (Dec-Jan-Feb) and autumn (Sep-Oct-Nov). On the contrary, during P2, the reversal into a negative correlation was statistically significant only during summer (Jun-Jul-Aug) with r = − 0.82, *p* < 0.01.

### Seasonal climatology and phenology

**Figure 3:**
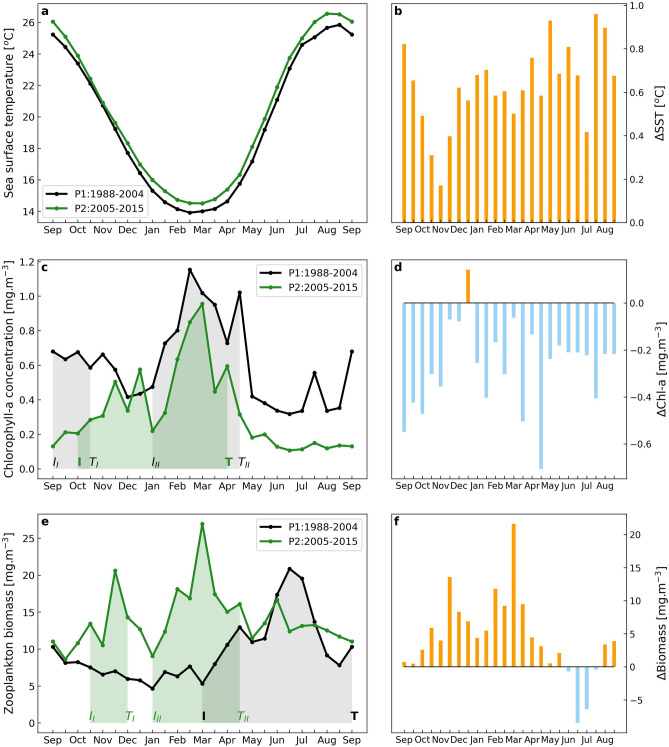
Biweekly climatological seasonal cycles for P1 (1988–2004) and P2 (2005–2015) and their differences [Δ, (P2–P1)] for SST (**a**, **b**), Chl-a (**c**, **d**) and zooplankton biomass (**e**, **f**), respectively. Phenology metrics are indicated by the shaded areas (duration of growth period) and annotated with a letter (I for timing of initiation, T for timing of termination), whereas a roman numeral (I, II) is added for distinction between multiple growth periods. Grey and light green coloring are used to indicate the P1 and P2 growth periods, respectively.

Seasonal cycles of SST, Chl-a and zooplankton biomass were investigated by estimating the biweekly climatologies over the periods P1 & P2, as well as their respective differences (P2–P1). Plankton phenological changes were examined by the observed shifts in phenology metrics of each study period (Fig. 3).

#### Sea surface temperature

The seasonal cycle of SST followed a similar pattern during both study periods, with SST minima observed during February and March and SST maxima during summer (August) (Fig. 3a). During P2, an overall increase in the mean biweekly SST values in comparison to P1 was evident, which reached approximately ~ 0.55 C° during February & March (maximum Chl-a concentrations) and ~ 0.8 C° during the warmer months (April–September, Fig. 3b).

#### Chl-a

During P1, three distinct phases of the Chl-a seasonal succession were observed during autumn, winter and summer (Fig. 3c). First, from the start of September [timing of initiation (*I*_*I*_)] until the second half of October [timing of termination (*T*_*I*_)], a period of moderate phytoplankton growth was evident (~ 6 weeks in duration), with the Chl-a peak (timing of maximum amplitude) occurring in the second half of September. During November and December, Chl-a gradually decreased. The main phytoplankton growing period lasted for ~ 16 weeks, initiating (*I*_*II*_) in early-January and terminating (*T*_*II*_) in late-April, whilst the Chl-a peak was observed in early-February, coinciding with the SST minima. Following the phytoplankton growth termination (*T*_*II*_), a period of low Chl-a concentration began and continued until the end of August, interrupted by a mild, transient increase in mid-July.

During P2, average Chl-a was lower in every month when compared to P1, except for December (Fig. 3c,d). Here, two main phases were identified in the seasonal cycle. The main winter phytoplankton growth period initiated in October and lasted until mid-April (*I to T*), with a duration of ~ 26 weeks. The non-growing period occurred in spring and summer, from mid-April to September. The timing of maximum amplitude occurred during the last two weeks of February, although a moderate increase was also evident during November–December, indicating a late-autumn phytoplankton growth.

#### Zooplankton biomass

An overall shift in zooplankton phenology was detected between P1 and P2 (Fig. 3e). During P1, zooplankton biomass remained low from mid-September to the end of February. The growth period initiated in early March (*I*) and continued for 26 weeks, until its termination in mid-September (*T*). Maximum zooplankton biomass occurred in the first half of June, when Chl-a exhibited its minima (Fig. 3c,e).

During P2, zooplankton biomass exhibited two separate growth periods, overlapping with the phytoplankton growth, one of mild intensity in late-autumn, which lasted for ~ 8 weeks from mid-October (*I*_*I*_) to mid-December (*T*_*I*_), and a second one of greater amplitude and duration, spanning 16 weeks from January (*I*_*II*_) to mid-April (*T*_*II*_) (Fig. 3e). The second zooplankton growth period reached its peak in early-March, 2 weeks after the Chl-a peak. A summer peak was still evident, although weaker than the one observed during P1 and with a shorter duration (June), and thus did not meet the criteria for a separate growth period. Zooplankton were characterized by higher biomass levels during P2, excluding a decrease in June and July (Fig. 3f).

### Zooplankton biomass variations in relation to S11 zooplankton abundance

**Figure 4:**
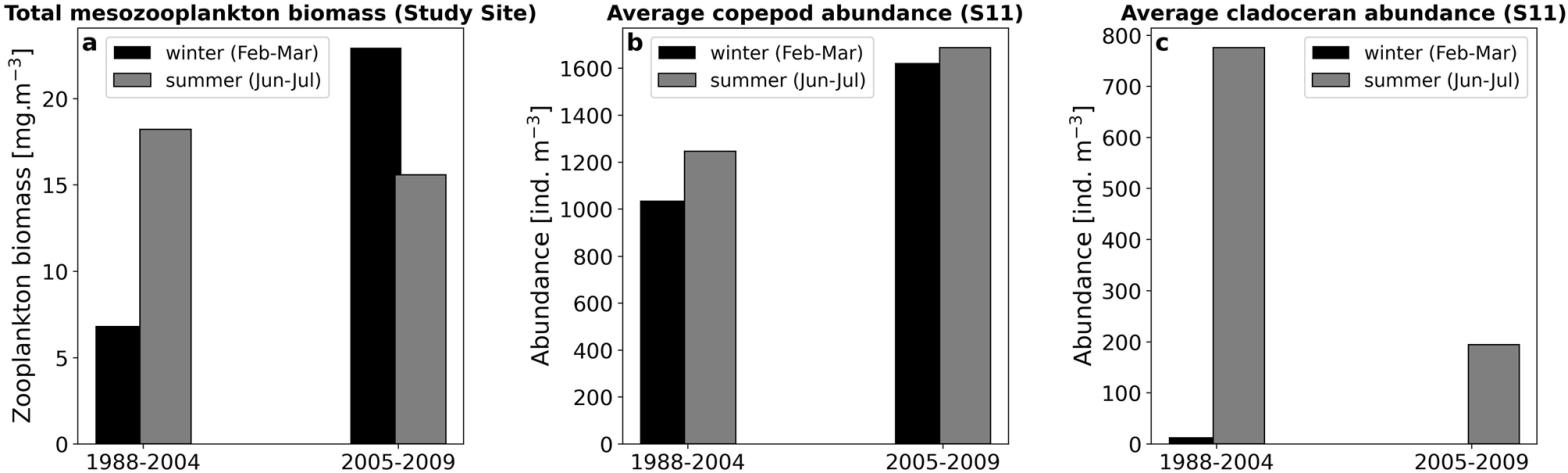
Total zooplankton biomass from the nearshore site of this study (**a**), average abundance of copepods at S11 station (**b**), and cladocerans at S11 station (**c**). The comparison is displayed in two periods: 1988–2004 and 2005–2009. Winter (black) is expressed as an average of values recorded in February and March, and summer (grey) as an average of June and July.

To further investigate the observed alterations in zooplankton biomass between the two study periods, an additional copepod and cladoceran abundance dataset from the offshore station S11 was utilized (Fig. 4). Due to the proximity between S11 and the study site, it was assumed that the copepod and cladoceran percentages were comparable to the variability in mesozooplankton biomass dataset. For each dataset, average values from February and March were used to represent the late winter peak in zooplankton biomass observed during P2, and average values for June and July were used to indicate the summer peak (mainly observed during P1). The increase in total zooplankton biomass during P2 (in comparison to P1) was also evident for average copepod abundances, with a ~ 56% and ~ 35% increase during winter (from 1035.2 to 1622.5 ind. m^−3^) and summer (from 1245 to 1687 ind. m^−3^), respectively. On the contrary, during winter, the average cladoceran abundance exhibited very low levels, close to zero (P1 & P2). Interestingly, similarly to the observed decrease in summer biomass during P2, a substantial reduction (~ 75%) in the summer average abundance of cladocerans (775.27 → 194 ind. m^−3^) was evident for 2005–2009.

## Discussion

The current study provides evidence of alterations in both plankton biomass and phenology in a nearshore ecosystem (Saronikos Gulf) of the Aegean Sea, under the influence of sea surface warming and previously reported shifts in the ecological quality status of the Saronikos Gulf^70,72,75,76,89,90^. The period 1989–2004 (P1) was characterized by lower SST, higher Chl-a concentrations and reduced mesozooplankton biomass (Fig. 2). In addition, a phenological asynchrony between phytoplankton (early autumn and winter growth periods) and zooplankton (summer growth) was evident (Fig. 3). In contrast, during 2005–2015 (P2), lower Chl-a concentrations were associated with higher zooplankton biomass and elevated SSTs. Zooplankton were negatively correlated with SST, especially during summer (Supplementary Fig. S3), while zooplankton summer growth decreased, and zooplankton phenology coincided with the main winter phytoplankton growth period.

### Long-term changes in nutrient concentrations and environmental status

Despite the lack of simultaneous nutrient data from the study site, a comprehensive understanding of the region's nutrient variability is available from the existing literature^71,72,75,91^. Previous research has described nutrient concentrations in the Inner Saronikos Gulf^75,90,91^. These studies, conducted across several sampling sites (see Fig. 1 in *Pavlidou *et al*.*^90^) at varying distances from the WWTP outfall to the open Aegean Sea, indicated improved ecological conditions with increasing distance from the sewage outfall. It is important to acknowledge that the observed improvements in water quality within the Saronikos Gulf have been attributed to the successful WWTP operations^70,73,76,77^.

Prior to the WWTP operation (1988–1994), zooplankton biomass remained lower, in the presence of higher Chl-a values (Fig. 2a), while both exhibited asynchronous seasonal timings (Fig. 3c,e), thus indicating a weak top-down control. During the primary stage of sewage treatment (1995–2004), an overall increase in nutrient concentrations occurred due to the increasing sewage volume, which was discharged at 60 m depth (below the thermocline)^75^. In parallel, Chl-a concentrations gradually decreased (0.1–0.4 mg m^−3^), indicating a shift to less mesotrophic conditions in the surface layer, whereas mesozooplankton biomass exhibited an increasing pattern from 1996 to 2006 (Fig. 2a). The reported Chl-a decline after 1999 is presumed to be driven by a combination of: a) lower surface nutrient concentrations, due to the increased depth of sewage discharge (60 m) after 1994^55,70^, b) an improvement in grazing control from mesozooplankton, and c) nutrient consumption by bacteria in deeper layers, facilitated by abundant concentrations of dissolved organic carbon (DOC)^74,92^. Similar interannual trends in Chl-a and mesozooplankton biomass (1987–2004) have also been observed at station S11^70^ (see their Fig. 3).

During P2 (2005–2014), WWTP improvements resulted in substantially lower nutrient concentrations and organic load^70,75,77,90^. Based on the nutrient concentrations reported in *Pavlidou *et al*.*^75^*,* the relative nutrient difference (%) was calculated between the periods 1995–2004 and 2005–2010 (see their Table 10.4). A decrease of ~ 44% in PO_4_, ~ 15% in SiO_4_, ~ 47% in NO_2_, ~ 38% in NO_3_, and ~ 30% in NH_4_ was reported at station S11, and similar trends were also observed in other monitoring stations of the Inner Saronikos Gulf^75^. Moreover, the trophic classification of station S11 shifted towards *lower mesotrophic* for phosphate and *oligotrophic* for nitrate between the periods 1995–2004 and 2005–2010 (see Table 10.5 in *Pavlidou *et al.^75^). Finally, based on Marine Strategy Framework Directive (MSFD) descriptors D1 (Biodiversity)^88^, D5 (Eutrophication) and D7 (Hydrography)^90^, the Inner Saronikos Gulf achieved a Good Environmental Status (GES) both spatially and temporally, moving from station S7 (sewage outfall) to S11 and from P1 to P2, respectively. Following this shift towards re-oligotrophication, there was a noticeable improvement in water quality^93,94^. Collectively, these changes played a pivotal role in shaping the results of this study, as zooplankton biomass reached higher levels, in parallel to increased Chl-a (although remaining lower than P1) in the nearshore study site (P2, 2005–2015), indicating a better grazing control on phytoplankton, contrary to P1 (Fig. 2a).

### Long-term warming in the Saronikos Gulf

An increasing trend in SST was identified throughout the time series (Supplementary Fig. S2), which is characteristic of the warming of the Mediterranean Sea^34,95–98^. A significant, negative interannual relationship was observed between Chl-a and SST (Fig. 2c). Although the Chl-a decrease was linked to the changes in the trophic status of the study area, more recent alterations to phytoplankton biomass and primary productivity (2015–present) remain to be characterized. At global scales, decreasing trends in phytoplankton biomass and primary productivity have been strongly correlated with warmer SSTs, tightly linked to the strengthening of water column stratification^99,100^. A time-dynamic Ecopath food-web model of the Saronikos Gulf, which explored the interacting impacts of fishing, climate warming and primary production^101^, showed that projected changes in primary production influenced the majority of the functional groups in the food web, through bottom-up control, while reduced productivity under more oligotrophic conditions resulted in decreased biomass and catches of many functional groups.

The metabolic rates of heterotrophic organisms are considered to be more impacted by warming, in comparison to photosynthetic rates, thus promoting grazing control over primary production^102–104^. Despite the zooplankton biomass increase and a more efficient grazing control on phytoplankton in P2, the shift from a positive relationship (P1) to a strong, significantly negative one (P2) between SST and zooplankton biomass suggested an opposite effect of warmer SSTs on zooplankton biomass (Fig. 2b). The timing of this switch, before and after the secondary stage of the WWTP, indicates an interplay between the effects of warming and changes in the ecological quality status of the Inner Saronikos Gulf. Under future warmer scenarios, climate-driven shifts in mesozooplankton biomass may ultimately occur in the Saronikos Gulf and other regional ecosystems in the Mediterranean. In the Gulf of Trieste (1995–1999), warmer temperatures drove a negative interannual shift in the correlation between temperature and copepod abundances, which occurred concurrently with a gradual, long-term reduction in inorganic phosphorus^105^. In the Central Atlantic, a ninefold decrease in mesoplankton biomass has been documented for the period 1950–2016, coupled with a significant SST increasing trend, Chl decline and an expansion of Chl-depleted areas^106^. Mesozooplankton’s negative correlation with SST was notably stronger during summer (P2) (Supplementary Fig. S3), paralleling the decrease in summer mesozooplankton biomass under higher temperatures, which was observed in the climatology of P2 (see further discussion in the section *Shifts in plankton phenology and dynamics*).

### Shifts in plankton phenology and dynamics

A phenological asynchrony between phytoplankton and zooplankton was detected during the first period (P1; Fig. 3c,e). Phytoplankton phenology was characterized by a main mid-winter/early spring growth period and a shorter one occurring in early autumn. The main phytoplankton growth during February–March is consistent with the typical seasonal growth of phytoplankton communities in the Mediterranean Sea, owing to the relatively high solar irradiance as well as to enhanced water column mixing conditions^32,107,108^. The occurrence of an autumn growth period has been linked with the erosion of the seasonal thermocline and higher surface nutrient concentrations due to increasing rainfall^59,86,109–111^. However, during P1, the initiation of the autumn growth observed in September (Fig. 3c, [I_I_]), occurred while the seasonal thermocline was considered to be strong and well-developed^69,75^. Thus, this earlier phytoplankton growth period could be partly explained by the human-induced, relatively high levels of nutrient concentrations that persisted throughout summer during the first study period^75^. Another factor contributing to the early autumn phytoplankton growth period could be the main zooplankton growth period in P1 (March–September), as zooplankton biomass maxima are usually accompanied by elevated excretion rates of dissolved nutrients (i.e., nitrogen and phosphorus), which can stimulate phytoplankton growth^112,113^. Previous studies in the coastal Saronikos Gulf, carried out during P1, have reported that zooplankton growth, excretion rates and metabolic activity were highest during summer, when cladocerans (e.g., *Penilia avirostris*) were the dominant taxa (up to 90% of total biomass). In addition, the absence of a correlation with Chl-a indicated the use of alternative food sources, other than phytoplankton, by zooplankton^109,114^.

At station S11, both copepod and cladoceran average abundances in P1 were high during summer (Fig. 4), possibly contributing to the main summer zooplankton biomass growth period. However, during P1, copepods maintained similar levels of abundance during February–March. Therefore, the initiation of zooplankton growth in March (Fig. 3e) could be associated with increased copepod abundance. In the Saronikos Gulf, copepods dominate mainly from October to May, whereas the coastal Inner part is characterized by the dominance of the copepod *Acartia clausi* in late-winter and spring^59,89^. The relatively high copepod abundance, regardless of season (Fig. 4), may be attributed to their adaptational flexibility towards environmental changes and fluctuations in food availability^115^, as well as to the seasonal variability and succession of different copepod species^116^.

During P2, a temporal match between phytoplankton and zooplankton phenology was found. A single, prolonged winter phytoplankton growth period was detected (Fig. 3c), characterized by a main Chl-a peak in February, which was common for both study periods. However, the timings of initiation and termination were ~ 4 weeks later and ~ 2 weeks earlier, respectively, in comparison to P1. The observed changes in phytoplankton phenology could be linked to warmer SSTs detected in P2 (Fig. 3b). Surface warming is able to cause an earlier establishment and prolonged duration of water column stratification, as shown in other Mediterranean coastal regions^117^, with the potential to limit nutrient redistribution into the euphotic zone, and subsequently alter the phenology of phytoplankton communities^5,25,118^. In response to oceanic warming, similar phenological shifts in diatoms and dinoflagellates have been reported in the western Baltic Sea^119^ and the Central North Sea^20^. The reported phenological shifts could also be linked to alterations in the phytoplankton community structure^120,121^. The effects of warming on Mediterranean coastal phytoplankton community composition have been investigated and indicate an increase in the abundance of picophytoplankton over larger taxa (such as diatoms), with increasing temperature^122–126^. The potential advantage of small phytoplankton species might lead to poorer feeding conditions for the zooplankton community and eventually to a decrease of the energy transfer to higher trophic levels^125,127^.

Following the phytoplankton seasonality in P2, zooplankton biomass exhibited an initial growth period from mid-October to mid-December, peaking in late-November and coincided with the moderate Chl-a increase in late-autumn (Fig. 3c,e). Similarly, the enhanced zooplankton growth from January–April was tightly linked to phytoplankton phenology and exhibited a maximum peak two weeks after the main Chl-a peak. The strong coupling between zooplankton and phytoplankton growth periods in P2 suggests a direct relationship between the two communities, through efficient grazing control. However, mesozooplankton exhibit diverse feeding habits, depending strongly on food availability. For example, copepods (e.g., *A. clausi* & *C. typicus*) and cladocerans (e.g., *P. avirostris*) are mostly filter feeders, preying preferentially on autotrophs and microzooplankton^128–130^. Alongside mesozooplankton, microzooplankton is considered to consume about two-thirds of total daily primary production, in many Mediterranean systems^129^. Therefore, both direct and indirect (preying upon microzooplankton) trophic links were assumed to connect mesozooplankton biomass/abundance with phytoplankton biomass.

The increase of average copepod abundance from P1 to P2 indicated that copepods were favored by the ecological conditions that prevailed during 2005–2015. The higher copepod abundance in P2 (Fig. 4b) might have contributed to the main zooplankton growth (Jan–Apr), as well as to the transient, mild increase observed during June (Fig. 3e). *Berline *et al.^92^ also reported an increase in the annual average abundances of copepods, appendicularians, and chaetognaths at station S11, over a period of elevated nutrient concentrations (1987–2004). A reduction in copepods’ community similarity has been reported over the period 1987–2007 in the Saronikos Gulf, further supporting potential changes in zooplankton community composition^131^.

In addition to the changes in trophic status, shifts in zooplankton phenology may be driven by increasing temperatures^132^. In the Central North Sea, the seasonal timing of peak biomass for copepods, decapods and other meroplankton and holozooplankton have all been occurring earlier, ranging from 2.2 to 10 days earlier per decade, or 11.1 to 52 days earlier per °C increase^7,20^. Similarly, in the Subarctic Pacific Ocean, oceanic warming has resulted in the peak biomass of the main copepod, *Neocalanus plumchrus*, occurring 14 days earlier per decade or 73 days earlier per °C increase^133^.

The decline in summer zooplankton biomass in P2 (Fig. 3e,f), compared to P1, paralleled the distinct reversal in the interannual correlation between zooplankton biomass and SST from a positive (P1) into a negative (P2) phase (Figs. 2; S3). Collectively, these results support the hypothesis that sea surface warming (2005–2015), especially during summer, did not favor zooplankton growth as it did during P1. The ~ 75% decrease in summer cladoceran abundance (2005–2009, Fig. 4) could account for the reduction in summer zooplankton biomass, observed during P2 (Fig. 3f). Despite being warm-water species^109^, cladoceran growth may be limited by extreme warming events, as was the case for *P. avirostris* in the northwest Mediterranean Sea^134^. In addition, high levels of cladoceran abundance have been associated with eutrophic waters, whereas in oligotrophic environments they exhibit much lower densities^134,135^. In the Inner Saronikos Gulf, during the more mesotrophic period 1987–1994, cladocerans exhibited a seven fold increase^92^. Conversely, with the transition to less mesotrophic and warmer conditions (between 1987–1994, 1995–2004, and 2005–2010) the most abundant cladoceran species, *Penilia avirostris*, saw a decrease in summer relative abundance^88^. Thus, both the SST warming trend and the prevalence of oligotrophic conditions after 2005^75,90^ are suggested as major drivers of the decline in summer mesozooplankton biomass and cladoceran abundances. The limited number of samples (Supplementary Fig. S5) available for the period 2005–2009 is a potential caveat for the zooplankton community abundance analysis, and thus, this result should be interpreted with caution. As current observations (Fig. 4c) were derived from two months of data per period (summer / winter), it cannot be excluded that potential shifts in cladoceran phenology may have taken place alongside rising temperatures, supporting the need for further investigation.

Here, documented changes in plankton biomass and phenology in the Saronikos Gulf were driven by an interplay of continuous oceanic warming and alterations in the ecological quality. Nevertheless, other factors than those included in the study could have played a role in these changes. For example, salinity changes due to climate warming may impact planktonic organisms (e.g., through changes in respiration, disruption of cellular processes) and their habitats (e.g., changes in water column stratification)^136^. However, an analysis of a salinity dataset acquired at the study site, as shown in Supplementary Fig. S4, did not indicate any connection between long-term or climatological variations in salinity and the observed plankton shifts reported in this study. Although wastewater management has improved in the Saronikos Gulf, increasing trends in sea surface warming will likely continue to impose impacts on plankton ecology^137,138^, including possible alterations to plankton community composition and structure. The spawning season of many Eastern Mediterranean fish stocks is highly dependent on phyto- and zooplankton seasonality^139^ and subsequent alterations to the spawning habits of exploited stocks may also occur due to warming^140,141^. The ecological consequences of the reported phenological shifts on higher trophic levels remain to be investigated.

### Supplementary Information

Supplementary Information.

## Data Availability

The data sets derived from the CMEMS Reprocessed (REP) Mediterranean (MED) SST product are available at: https://data.marine.copernicus.eu/product/SST_MED_SST_L4_REP_OBSERVATIONS_010_021/description.
