# Supplementary material for: Long-term warming and human-induced plankton shifts at a coastal Eastern Mediterranean site
Source: Sci Rep. 2023 Nov 29;13:21068. doi: 10.1038/s41598-023-48254-7 (PMC10687065; doi:10.1038/s41598-023-48254-7)
Supplement: Supplementary file 1 — Supplementary Information. [file 41598_2023_48254_MOESM1_ESM.pdf]

Long-term warming and human-induced plankton shifts at a coastal Eastern Mediterranean site

Kalloniati K.[1*], Christou E.D.[2], Kournopoulou A.[1], Gittings J.A.[1], Theodorou I.[1], Zervoudaki S.[2], & Raitsos D.E.[1]

[1]Department of Biology, National and Kapodistrian University of Athens, Athens 15772, Greece

[2]Institute of Oceanography, Hellenic Centre for Marine Research (HCMR), Anavyssos 19013, Attica, Greece

SUPPLEMENTARY DATA

Table S1: Table of Pearson's correlation coefficients (r) and p-values for P1 (1989-2004; N=16), P2 (2005-2014; N=10) and the total period (P1+P2, 1989-2014; N=26). The top rows of each table exhibit the correlation coefficients (r) and respective p-values which characterize the interannual trends (of zooplankton biomass, Chl-a and SST) over each of the time periods. The following rows show the correlation coefficients (r) and p-values between zooplankton biomass, Chl-a, and SST, again, during each of the study periods, all based on annual mean values. The statistically significant p-values (<0.05) are indicated in bold.

**Period 1 (P1)**	Zooplankton biomass	Chlorophyll Concentration	SST
Years (Trend)	0.83, p<**0.0001**	-0.80, **0.0001**	0.73, **0.0007**
Zooplankton biomass	—	-0.71, **0.0012**	0.85, p<**0.0001**
Chlorophyll Concentration	-0.71, **0.0012**	—	-0.51, **0.003**
SST	0.85, p<**0.0001**	-0.51, **0.003**	—

**Period 2 (P2)**	Zooplankton biomass	Chlorophyll Concentration	SST
Years (Trend)	-0.33, p>0.05	0.15, p>0.05	0.71, **0.0316**
Zooplankton biomass	—	-0.66, **0.0498**	-0.66, **0.0486**
Chlorophyll Concentration	-0.66, **0.0498**	—	0.25, p>0.05
SST	-0.66, **0.0486**	0.25, p>0.05	—

**Whole period (P1+P2)**	Zooplankton biomass	Chlorophyll Concentration	SST
Years (Trend)	0.62, **0.0006**	-0.74, p<**0.0001**	0.79, p<**0.0001**
Zooplankton biomass	—	-0.71, p<**0.0001**	0.46, **0.0179**
Chlorophyll Concentration	-0.71, p<**0.0001**	—	-0.52, **0.0059**

SST	0.46, **0.0179**	-0.52, **0.0059**	—

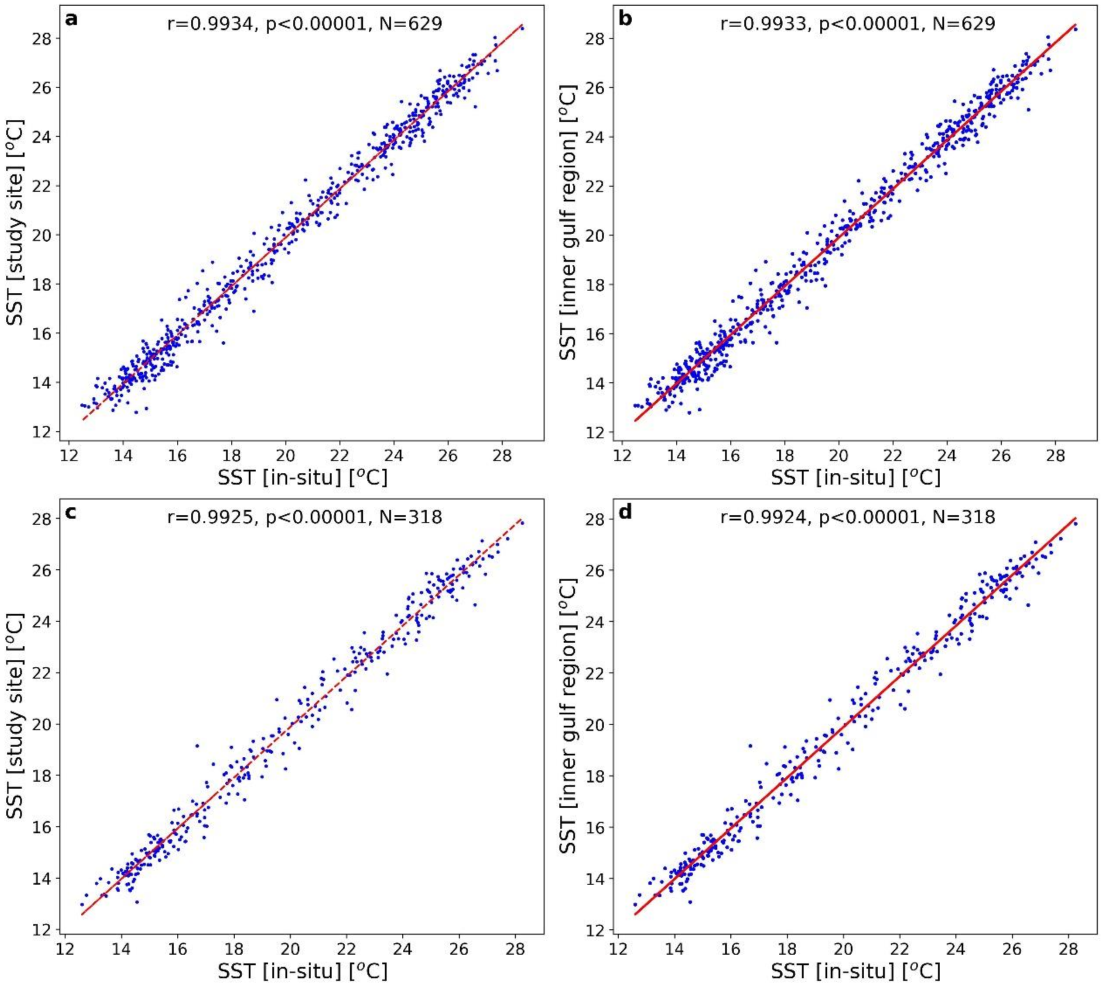

Figure S1: Comparison between the *in-situ* SST data set and the CMEMS reprocessed (REP) Mediterranean (MED) SST data set, for the period Nov/1988 – Apr/2015: Daily match-up between the biweekly *in-situ* SST time series and the remotely-sensed SST time series, extracted from 1 pixel centered above the study site (37.89N, 23.71E) (a), and (b) spatially averaged over a region of the inner Saronikos gulf, including the study site and station S11 (37.8, 37.92N, 23.60, 23.74E). Pearson's correlation coefficient analysis between the monthly mean values of the *in-situ* and remotely sensed data sets (1 pixel above study site – (c), and inner Saronikos Gulf region – (d)).

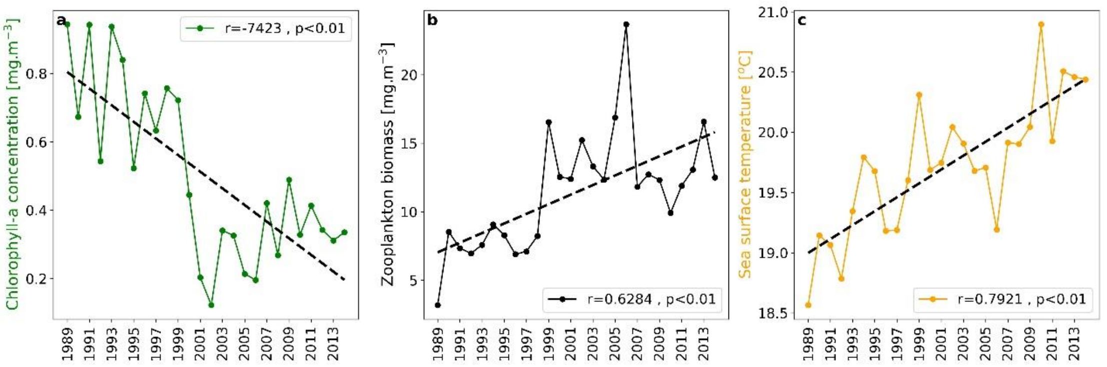

Figure S2: Interannual trends of Chlorophyll-a concentration (a), Zooplankton biomass (b) and Sea surface temperature (c), based on annual averages, for the period 1989-2014.

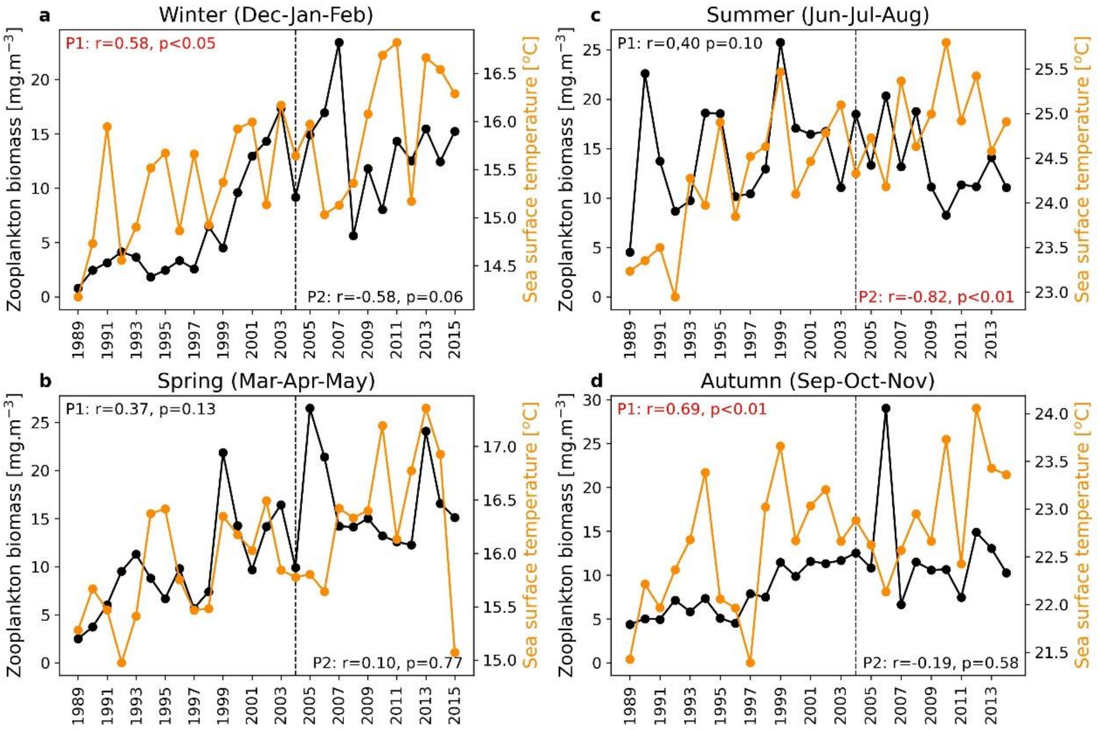

Figure S3: Interannual variability of zooplankton biomass versus sea surface temperature, during winter ([a], December – January – February), spring ([b], March – April – May), summer ([c], June – July – August) and autumn ([d], September – October – November). Each value depicts the average of the biweekly values of each trimester.

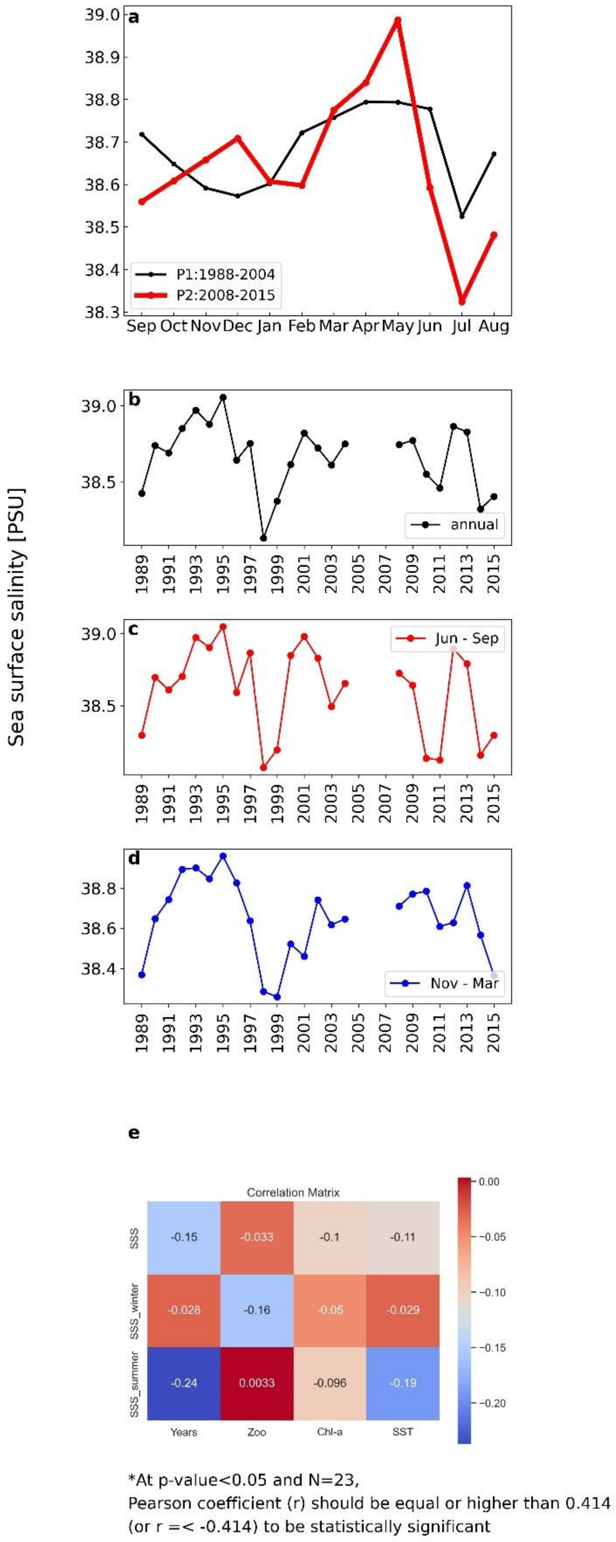

Figure S4: Monthly sea surface salinity (SSS) climatology for each of the study periods (P1, P2) (a) and interannual variability for the period 1989-2015, based on annual mean values (b), average values from June to September ("summer period") (c) and average values from November to March ("winter period"), (d). Concerning the differences in climatology between the two study periods (a), an independent t-test was performed for P1 and P2 climatologies and no significant difference was identified (T-statistic: 0.64, p-value: 0.52). In panel (e) there is a Pearson's correlation matrix, showing that there are neither any statistically significant (p<0.05) salinity trends (SSS vs. years)

nor interannual correlations with Zooplankton biomass, Chl-a, and SST. Salinity data are missing from the years 2005, 2006 and 2007.

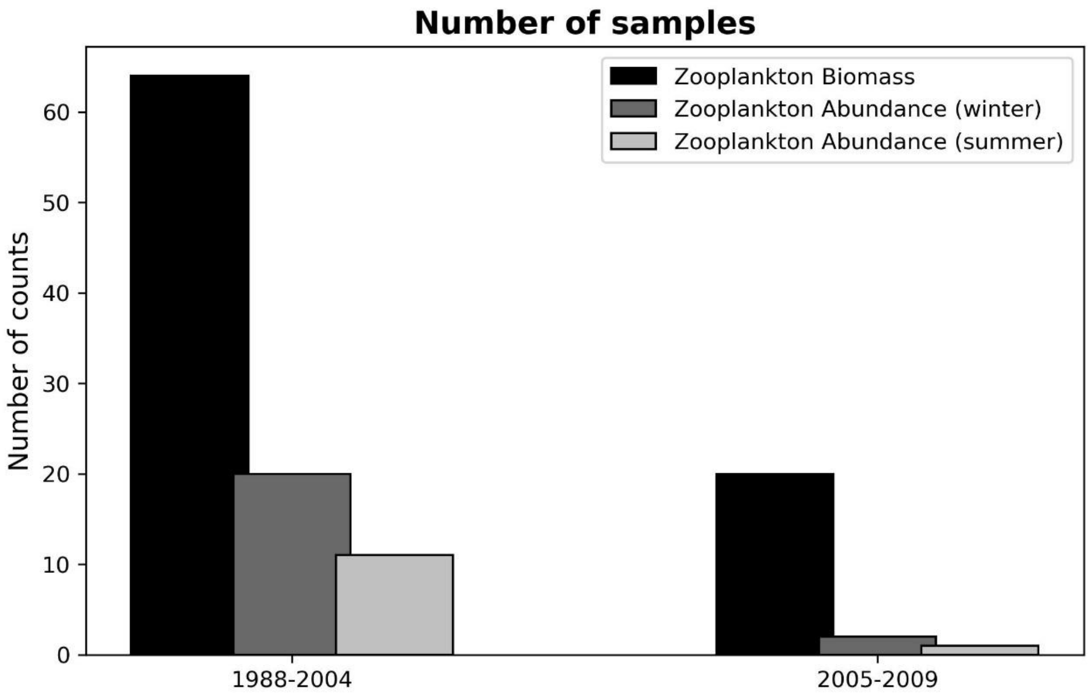

Figure S5: Number of samples used for comparison of the changes in zooplankton biomass peaks, between the periods 1988-2004 and 2005-2009, with the changes in abundance of the most representative zooplankton groups in the area, copepods and cladocerans, during the same periods (depicted in Figure 5).
